# A multimodal virtual vision platform as a next-generation vision system for a surgical robot

**DOI:** 10.1007/s11517-024-03030-1

**Published:** 2024-02-02

**Authors:** Young Gyun Kim, Jong Hyeon Lee, Jae Woo Shim, Wounsuk Rhee, Byeong Soo Kim, Dan Yoon, Min Jung Kim, Ji Won Park, Chang Wook Jeong, Han-Kwang Yang, Minwoo Cho, Sungwan Kim

**Affiliations:** 1 Interdisciplinary Program in Bioengineering, Seoul National University, 1 Gwanak-Ro, Gwanak-Gu, Seoul, 08826 Republic of Korea; 2 Seoul National University Hospital, 101 Daehak-Ro, Jongno-Gu, Seoul, 03080 Republic of Korea; 3 Department of Surgery, Seoul National University College of Medicine, 103 Daehak-Ro, Jongno-Gu, Seoul, 03080 Republic of Korea; 4 Department of Urology, Seoul National University College of Medicine, 103 Daehak-Ro, Jongno-Gu, Seoul, 03080 Republic of Korea; 5 Department of Transdisciplinary Medicine, Seoul National University Hospital, 101 Daehak-Ro, Jongno-Gu, Seoul, 03080 Republic of Korea; 6 Department of Medicine, Seoul National University College of Medicine, 103 Daehak-Ro, Jongno-Gu, Seoul, 03080 Republic of Korea; 7 Department of Biomedical Engineering, Seoul National University College of Medicine, 103 Daehak-Ro, Jongno-Gu, Seoul, 03080 Republic of Korea; 8 Artificial Intelligence Institute, Seoul National University, 1 Gwanak-Ro, Gwanak-Gu, Seoul, 08826 Republic of Korea

**Keywords:** da Vinci research kit, Head-mounted display, Virtual vision platform, Usability, User evaluation

## Abstract

Robot-assisted surgery platforms are utilized globally thanks to their stereoscopic vision systems and enhanced functional assistance. However, the necessity of ergonomic improvement for their use by surgeons has been increased. In surgical robots, issues with chronic fatigue exist owing to the fixed posture of the conventional stereo viewer (SV) vision system. A head-mounted display was adopted to alleviate the inconvenience, and a virtual vision platform (VVP) is proposed in this study. The VVP can provide various critical data, including medical images, vital signs, and patient records, in three-dimensional virtual reality space so that users can access medical information simultaneously. An availability of the VVP was investigated based on various user evaluations by surgeons and novices, who executed the given tasks and answered questionnaires. The performances of the SV and VVP were not significantly different; however, the craniovertebral angle of the VVP was 16.35° higher on average than that of the SV. Survey results regarding the VVP were positive; participants indicated that the optimal number of displays was six, preferring the 2 × 3 array. Reflecting the tendencies, the VVP can be a neoconceptual candidate to be customized for medical use, which opens a new prospect in a next-generation surgical robot.

**Graphical Abstract:**

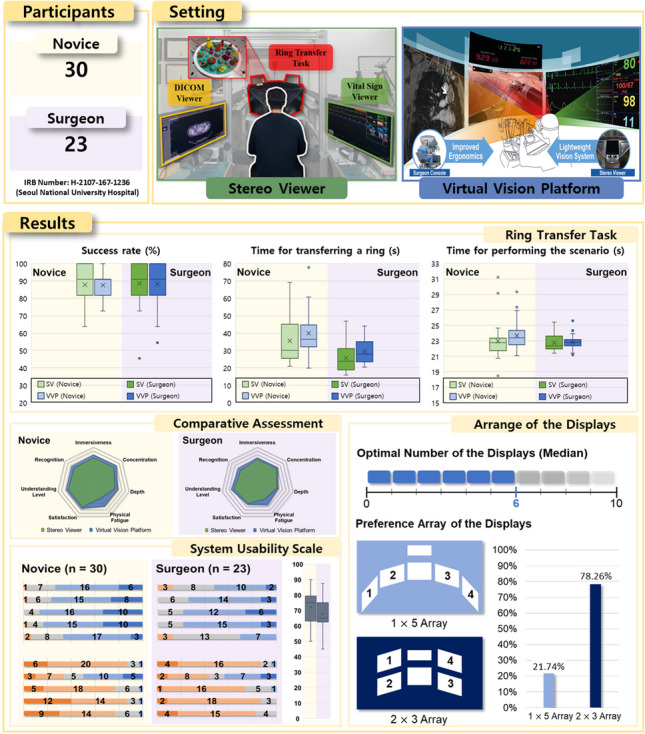

**Supplementary Information:**

The online version contains supplementary material available at 10.1007/s11517-024-03030-1.

## Introduction

Minimally invasive surgery (MIS) is an alternative to open surgery owing to the enhanced recovery caused by small incisions and minimal tissue manipulation [1–3]. However, it needs improvements to alleviate the movement constraints within the confined surgical field, such as the abdominal cavity [3]. Robot-assisted surgery (RAS) overcomes the disadvantages of MIS, including the restricted surgical view and instrumental movement in the abdomen [4–6]. It provides surgeons with a three-dimensional vision and enhanced functional assistance. Therefore, the utilization of RAS has increased rapidly and broadened the adoption range in the surgical field for decades [5–7]; accordingly, the necessity for ergonomic improvement in these systems for their use by surgeons has been increasing.

The da Vinci surgical system (dVSS, Intuitive Surgical, Inc., Sunnyvale, CA, USA), the most representative RAS platform, has gained global popularity among surgeons and patients owing to its stereoscopic vision system, ergonomic manipulation, decreased blood loss, and shorter hospital patient stays compared with those of laparoscopic surgery [7–9]. Owing to its acceptable feasibility and safety, the dVSS has been applied in diverse medical fields, such as urologic, colorectal, gynecologic, and gastrointestinal surgeries; and numerous surgeons expect exponential growth in dVSS applications [10–14]. However, surgeons continue to request ergonomic improvements owing to issues such as work-related musculoskeletal fatigue on their necks, shoulders, and backs induced by prolonged dVSS use, and intrinsic barriers to console-to-bedside communication [14–17]. The structure of the conventional vision system forces the operator to lower the head to see the surgical site [18]. Therefore, previous research has attempted to eliminate the restrictions on the user’s posture [19–22].

Based on the advancement of virtual reality (VR), augmented reality, and mixed reality technologies, diverse applications of the head-mounted display (HMD) in medicine have been investigated [23–25]. Among the neoconceptual HMDs, the VR HMD has emerged as an innovative tool because it can provide an immersive and interactive environment to the user [25, 26]. Furthermore, there have been positive responses to the VR HMD regarding effectiveness, efficiency, and satisfaction based on its usability [26–29]. Another ergonomic advantage of the VR HMD was discovered in some trials when applying it to the RAS platform [30]. The results confirmed that the VR HMD could be a candidate for the next-generation vision system of the RAS platform [30, 31]. However, it has some disadvantages: (i) the user physically sees a restricted surgical view through the binocular screens of the VR HMD; and (ii) the user must remove the VR HMD repeatedly to interact with the external surroundings [32, 33]. Additionally, there have been some issues with the conventional vision system. Checking other visual information during a robotic operation while wearing the HMD becomes challenging. To overcome these issues, the VR space was utilized in the present study to provide various types of medical information while wearing the HMD.

**Fig. 1:**
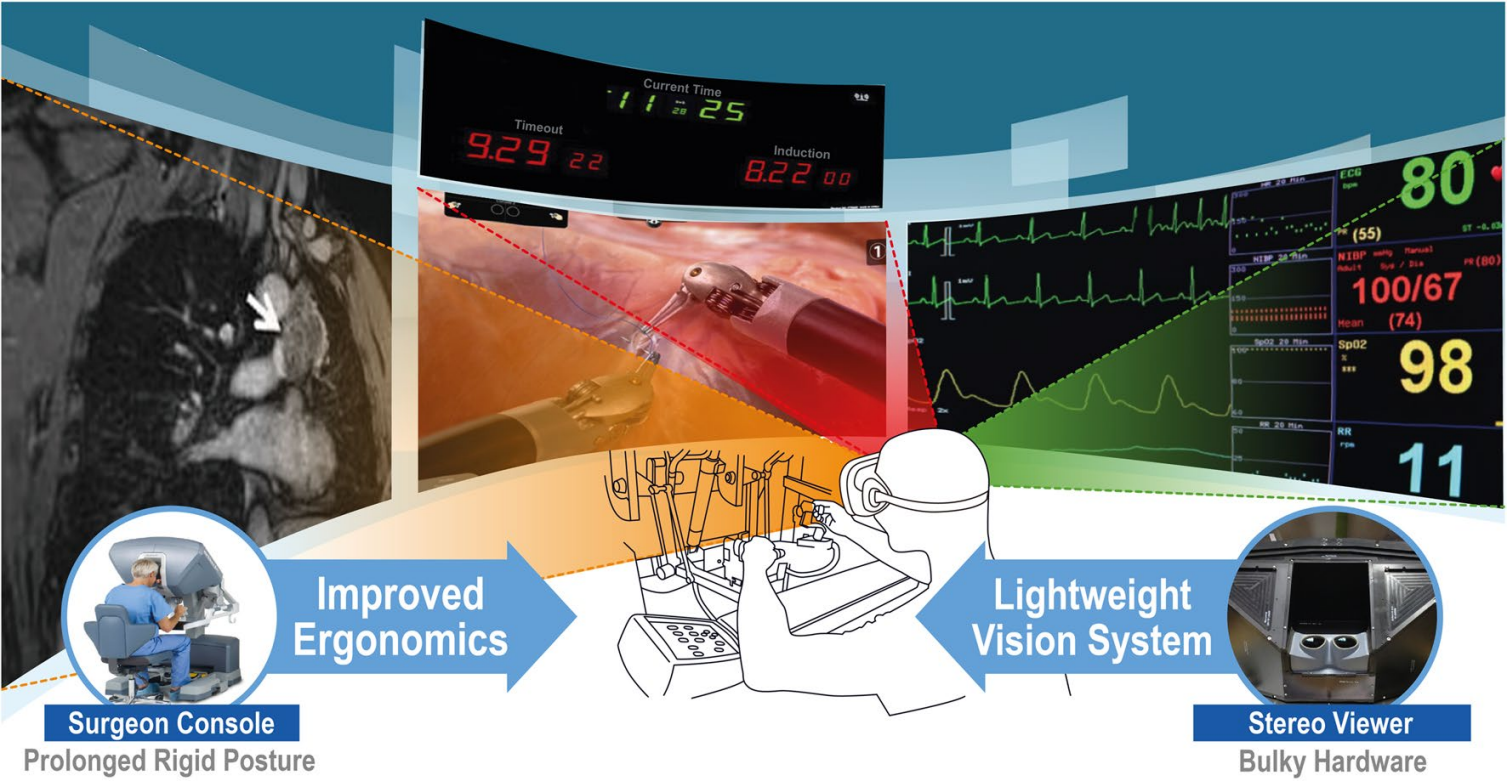
Conceptual diagram of the virtual vision platform (VVP). The middle image is the surgical view and timer, the left image is the digital imaging communication in medicine (DICOM) viewer, and the right image is the vital sign viewer

The present study proposes a novel candidate based on the VR HMD, called a virtual vision platform (VVP), to deal with the points of improvement, as illustrated in Fig. 1. The VVP provides various medical information in the VR environment on the screens of the VR HMD. It contains diverse types of medical data, such as those produced by computed tomography, magnetic resonance imaging, electronic medical records (EMR), and graphical and numerical information based on the patient monitor system. This can facilitate visual feedback more efficiently based on multiple screens. The surgical operating environment setting utilizing the VR HMD may be customized based on the preferences of individual users. Furthermore, the user can have an external view without removing the VR HMD. To investigate the VVP, a user evaluation for subjects, including clinical professors, fellows, residents, and novices, was performed based on the da Vinci research kit (dVRK, Intuitive Surgical, Inc., Sunnyvale, CA, USA) in an environment similar to the operating room environment, as in previous studies [20–22, 34, 35]. By analyzing each participant’s performance results and interviews, the present study aims to demonstrate the usability of the VVP as the ergonomically improved vision system of the RAS platform.

## Methods

### Preparation

#### da Vinci research kit

**Fig. 2:**
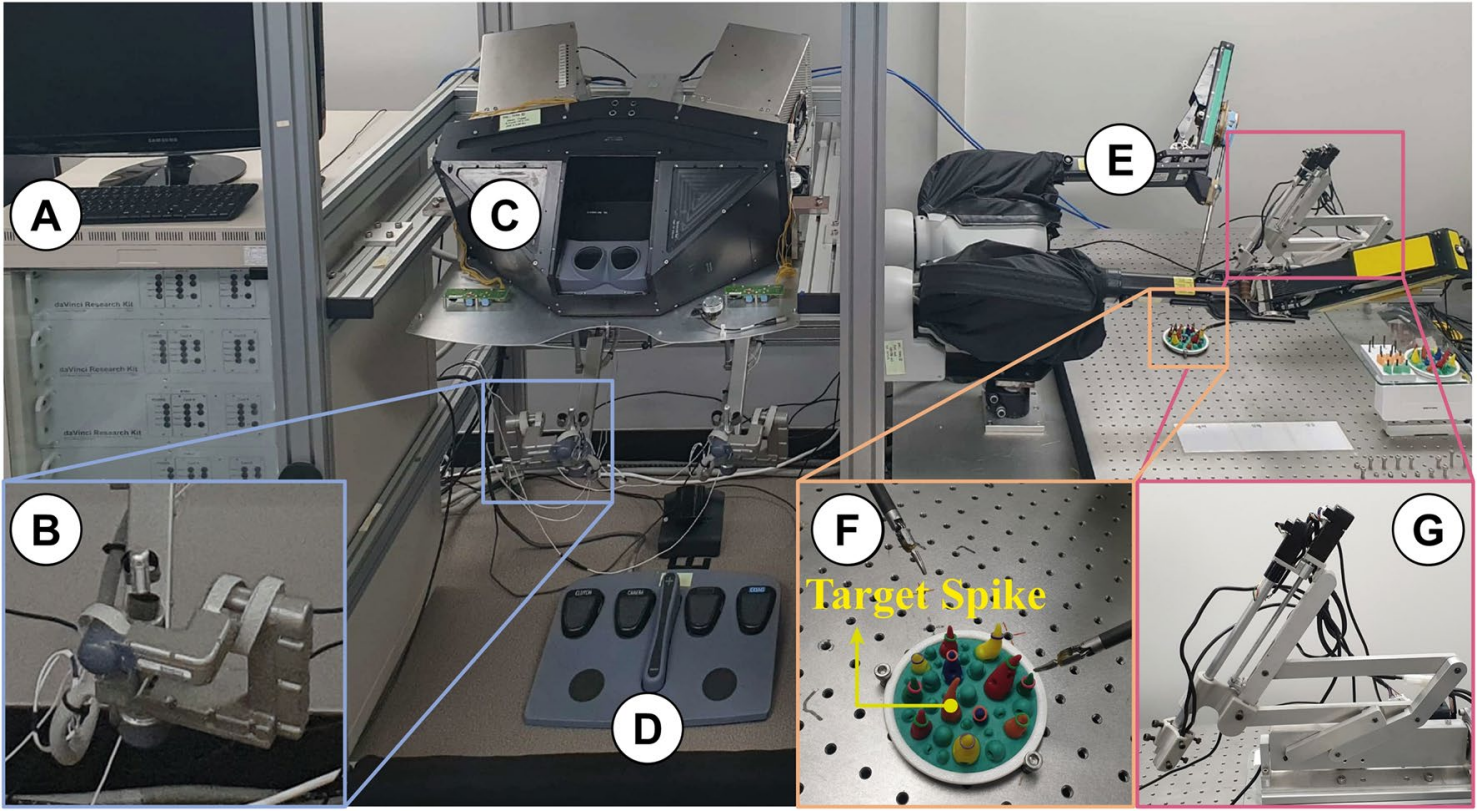
da Vinci research kit (dVRK). **A** 8-axis motor control units. **B** Master tool manipulators (MTMs). **C** Stereo viewer (SV). **D** Foot pedal tray. **E** Patient side manipulators (PSMs). **F** Sea spikes pod. **G** Endoscopic control system

The dVRK is an open-source telerobotic research platform based on the first-generation dVSS developed by Intuitive Surgical, Inc. [36]. It is a primary–secondary system divided into a surgeon console and patient-side robot. The surgeon console is composed of a pair of master tool manipulators (MTMs), a foot pedal tray with four pedals, and a stereo viewer (SV). The patient-side robot includes two patient side manipulators (PSMs), as shown in Fig. 2 [37]. As the user grabs and manipulates the MTMs to operate and perform the experimental task, the movement is transformed into a digital signal by an 8-axis motor control unit. Then, the motion data are scaled by a personal computer configured for the control and sent to the corresponding PSM. It consequently clones the movement of the MTMs at the end-effector side of the instrument mounted on each PSM. To view the end-effectors’ movements remotely on PSMs, a 4-degree of freedom (DOF) endoscopic control system with a maximum resolution of 1920 × 1080 pixels, which operates as a fulcrum point motion system, is utilized for user evaluation [20–22, 35]. After the processes of calibration and rectification with OpenCV libraries are completed, the stereoscopic video is displayed in the vision system using the SV or HMD.

#### Virtual vision platform

In our study, the HTC Vive Pro Eye (HTC Corporation, Taoyuan, Taiwan), which has a resolution of 1440 × 1600 pixels per eye, field of view of 110°, and a sampling rate of 90 Hz, was adopted as the main HMD model to develop the VVP framework. The VVP was designed using the Unity engine (Version 2019.1.10f1, Unity Technologies, San Francisco, CA, USA) to maximize convenience while using the HMDs by allowing users to see multiple displays inside the virtual environment, as illustrated in Fig. 1. To transmit the medical information to the displays, a medical image viewer, an EMR document viewer, a vital sign monitor, and various other types of software that can potentially aid the surgeon during an operation were utilized. Furthermore, inside the VR space built using the Unity environment, a user interface was designed to enable the user to add, select, position, and resize the displays, and allowed the user to choose the preferred information to be shown on each display, thus improving the flexibility and customizability of the interface.

#### Participants

The study was approved by the institutional review board (IRB) of Seoul National University Hospital (IRB No. H-2107–167-1236). In total, 53 participants were involved in the present study, including 23 surgeons and 30 novices who were not experts in the medicine. Among the surgeons, three clinical professors, seven fellows, and 13 residents participated in the user evaluation; the clinical professors and fellows are experts in robotic surgery, specializing in general and urological surgery. All participants followed the protocol after guidance was provided on the precautions for the safe manipulation of the dVRK.

### Study design

#### Overall flow

**Fig. 3:**
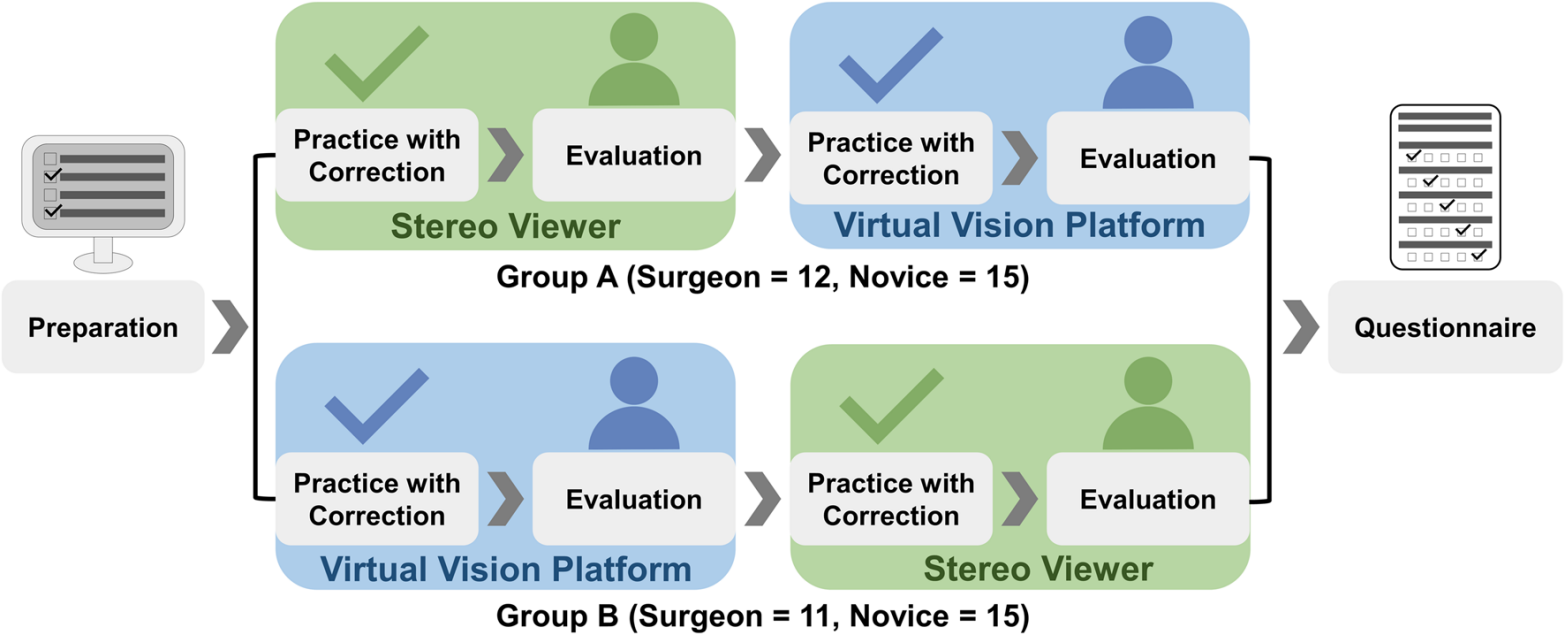
Overall flow of the user evaluation protocol

The protocol consisted of preparation, evaluations with SV and VVP, and questionnaire responses, as illustrated in Fig. 3. During the preparation, oral and visual explanations about the evaluation, and precautions associated with the manipulation of the dVRK were provided to the participants. The participants were divided into two random groups to prevent learning effects (induced owing to the preceded task) based on their experiences, according to a within-subjects design. Therefore, half of the participants practiced and performed the tasks in the SV environment first, while the other half performed the tasks in the VVP environment. Before conducting the task with the secondary vision system, a break was allowed for all participants to maintain similar test conditions and prevent them from being exhausted by the primary vision system. After the tasks with both vision systems, the participants were asked to respond to the questionnaire, based on their experience about the use of the SV and VVP. As an additional step, experimenters gave a demonstration to the surgeons on the medical information that they preferred to place at specific locations. Feedback was provided until the surgeons were satisfied with the arrangement. Subsequently, the experimenter recorded the optimal disposition tendency to suggest the standardized formation in the VR world.

#### Ring transfer task

**Fig. 4:**
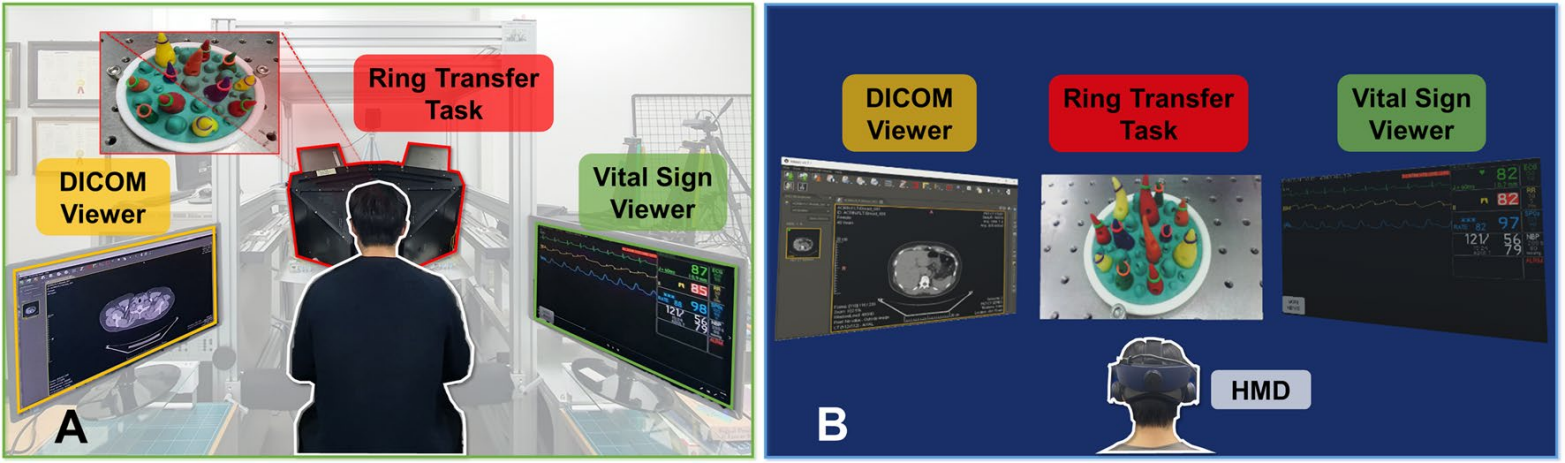
Experimental environment settings. **A** SV. **B** VVP

The ring transfer task (RTT) was performed using the SV and VVP with the surgeon and novice groups to investigate the feasibility of the VVP. Based on the sea spikes pod in Fig. 2F, the RTT—according to which the users move the rubber ring between the spikes—was performed repeatedly and can be transformed in diverse ways to accord with the purpose of the research [38–40]. To investigate the performance when using the SV and VVP, identical conditions except for the vision system were given to the participants, as shown in Fig. 4. The participants could execute the RTT by viewing the real-time scene on the middle section of the display system while simultaneously checking the DICOM viewer on the left and the vital sign viewer on the right by merely turning their heads. The participants were guided to manipulate the dVRK to transfer the 11 rings on each spike to the orange spike shown as “Target Spike” in Fig. 2F, viewing the display of their own performance of the task on the sea spike pod in real-time, as in previous research [41–43]. During the transfer of the ring, the participants were instructed to control the right and left MTMs on dVRK. For example, if a participant grasped a ring with the left MTM and transferred it to the opposite instrument mounted on the right MTM, the ring was then placed on the spike with the right MTM. There was no preferential order in which rings had to be transferred.

The success rate for transferring a ring to the target spike and the time required to transfer one independent ring were measured by the experimenter. The criteria for the time to transfer a ring was measured from the time the participants started to manipulate the dVRK to the time the ring was moved from the instrument and located entirely on the spike. The processes of all participants were recorded on video so that the experimenter could review the results by assessing the video frames and double-checking the transfer time. In addition, the craniovertebral angle (CVA), an indicator used to measure the total forward curvature of the back, was estimated to analyze the participants’ postures ergonomically [44, 45]. Before performing the RTT, the experimenter attached stickers to the three points, including the seventh cervical vertebra and tragus of the ear, to calculate the CVA. After the RTT, the experimenter extracted the frames every 2 s from the recorded videos of all participants, annotated the three major points, and calculated the CVA of each frame based on the coordinates of the annotated points.

#### Scenarios

Two different types of scenarios were assigned to the participants while performing the RTT to investigate the responses about the assumed circumstance of having to check the medical information of the patient: DICOM viewer check (DVC) and vital sign check (VSC). The scenarios were designed to be conducted by both novices and surgeons, regardless of their differences in medical expertise. In the DVC scenario, a participant identifies the information on the DICOM viewer. Specifically, the participants were asked to enlarge the medical images in the DICOM viewer. They were asked to speak five letters hidden in the image randomly. In the VSC scenario, the participants had to check numerical data on the vital sign viewer. Specifically, the participants were required to announce one numerical value among the multiple vital signs recorded according to the supervisor’s guidance: heart rate, respiratory rate, and oxygen saturation. If the alarm that notified the onset of the scenario rang during the execution of the RTT, the participants had to stop any ongoing process immediately and execute the given scenario.

The participants did not know when the alarm would ring. Both scenarios were designed to apply to all participants for general purposes. If the participants completed the respective scenarios, the supervisor terminated the execution scenario. The completion time of each scenario was measured from the sounding of the alarm to the termination announcement; these times were reviewed and double-checked by the experimenter based on the recorded videos.

#### Questionnaire response

Based on the individual experience of performing the RTT, all participants were required to respond to various questionnaires, including van der Laan’s technology acceptance score, system usability scale (SUS), the NASA task load index (TLX), and comparative assessment, to investigate the usability of the VVP. Van der Laan’s technology acceptance score is a standardized qualitative assessment of the degree of acceptance of newly conceptualized technology [46, 47]. It consists of two key indicators: usefulness (five sub-indicators) and satisfaction (four sub-indicators). It uses a 5-point Likert scale ranging from − 2 to + 2. The SUS is a metric composed of validated questions whose answers are utilized to measure a qualitative score about the usability of the new system. It comprises a 5-point Likert scale ranging from + 1 (strongly disagree) to + 5 (strongly agree) [48–50]. The NASA TLX is an extensively used method for estimating the workload across various indicators, such as mental demand, physical demand, temporal demand, overall performance, effort, and frustration. It is based on a 10-point Likert scale from + 1 (very low, perfect) to + 10 (very high, failure) [51, 52]. Finally, based on previous research on RAS implementation, the comparative assessment compared the environments of the stereo viewer and VVP with a 10-point Likert scale from + 1 (strongly unsatisfied) to + 10 (strongly satisfied) [53–56]. In addition, the participants were interviewed comprehensively about the vision system.

#### Optimal arrangement of multiple medical displays

In this section, the investigation of the optimal arrangement of multiple medical displays is implemented for the surgeons based on their clinical experiences. As shown in Fig. 8, the number of displays for the VVP was extended from four to six to adopt diverse types of medical information. The candidates for the medical information were captured image, vital sign viewer, DICOM viewer, and EMR. The surgeons were instructed to select the preferred medical information for each section, except for the middle part of the VVP where the performing surgical operation image and surgical timer were fixed. The experimenters subsequently gave a demonstration to the surgeons based on their preferred selection and made modifications until the surgeons were satisfied with the formation. The survey of the most appropriate arrangement of multiple displays, each representing the aforementioned information for the surgeon, comprehensively investigated factors such as the possibility of standardization, arrangement preference, medical information priority, and the number of displays that the surgeon could view without burden.

### Statistical analysis

Based on the overall flow of the user evaluation protocol, there were comparison cases between the SV and VVP in their RTT performance with scenarios and questionnaire responses. In the RTT, only CVA was analyzed using the independent sample *t* test. In the cases of the success rate and times required to transfer a ring, the statistical analysis procedure was omitted because it was difficult to expect significant results solely based on the differences in vision systems. The same concept applied to the time needed to perform the scenario operations. In the questionnaire responses, there was no statistical analysis of van der Laan’s technology acceptance score and SUS because these results were obtained only from the VVP, and there was no need to set the hypothesis to compare findings from the surgeons and novice participants. The independent sample *t* test and Mann–Whitney *U* test between the SV and VVP were conducted to analyze the results from the NASA TLX and comparative assessments, depending on whether normality and equal variance criteria were satisfied. In the case of the Mann–Whitney *U* test, the asterisk (*) was attached to the *p* value. All statistical analyses were conducted using the statistical package for the social sciences (SPSS 26.0, IBM, Armonk, NY, USA).

## Results

### Ring transfer task with scenarios

**Fig. 5:**
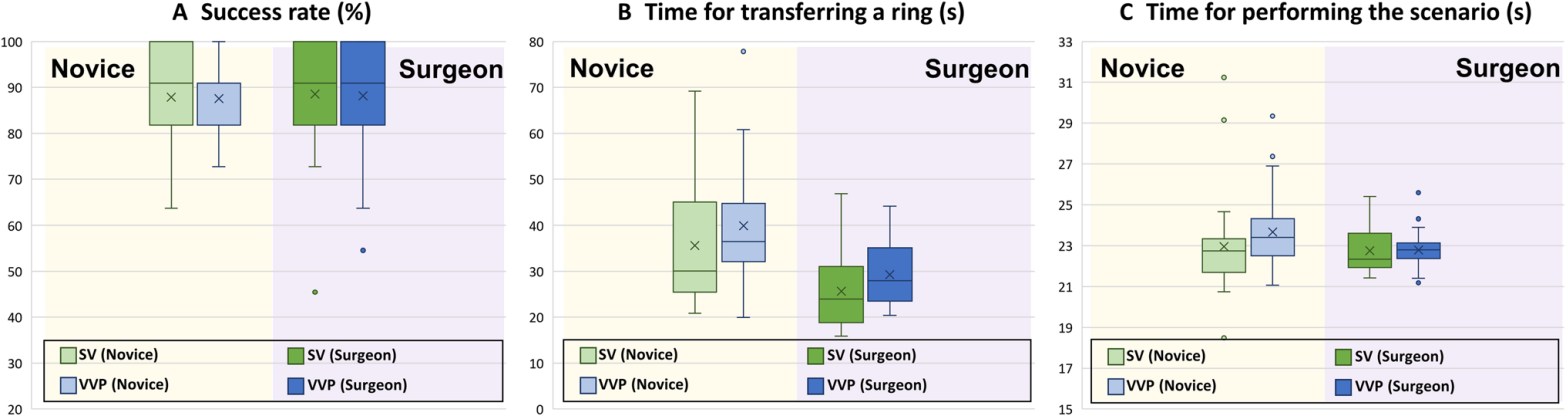
Results of ring transfer task in conjunction with the execution of various scenarios

In the RTT, the results for the success rate (measured by the number of rings transferred without being dropped) and the time required to transfer a ring and perform the scenario are presented in Fig. 5. In the success rate, there were differences between the SV and VVP, which were 0.30% and 0.40% in the novice and surgeon group cases, respectively. The standard deviation of the VVP was lower than the SV; specifically, the differences were 1.73% in the novice group and 0.80% in the surgeon group. Regarding the time required to transfer a ring, the average differences between the SV and VVP were 4.34 s and 3.57 s in the novice and surgeon group cases, respectively. The surgeons moved a ring faster than the novices, requiring 9.92 s using in the SV and 10.69 s using the VVP. The standard deviation of the VVP was 1.50 s lower than that of the SV in the novice group, and 0.49 s higher in the surgeon group. The average times required to complete the DVC and VSC scenarios are shown in Fig. 5C. The differences in the average time were 0.71 s and 0.03 s in the novice and surgeon groups, respectively. As shown in Fig. 5B and C, there was a tendency for fewer outliers in the surgeon than in the novice group, which is attributed to the familiarity of the surgeons with the RAS platform. The detailed data are shown in supplementary materials.

When conducting the RTT with the tested vision platforms, the CVA of all participants was calculated and compared between the SV and VVP, as listed in Table 1. The CVA results between the SV and VVP were significantly different in both groups (*p* < 0.05). This finding indicates that users can lower their heads by a smaller angle in the VVP than in the SV case. Owing to the extended number of DOF of the head in the cases in which the participants used the VVP, the standard deviation in the VVP was higher than that in the SV case.

**Table 1:** CVA measurements

Group	Vision system	Craniovertebral angle (°)		
		Mean	SD	*p* value
Novice (*n* = 30)	SV	43.68	2.25	< 0.05
	VVP	59.47	3.61	
Surgeon (*n* = 23)	SV	40.23	1.67	< 0.05
	VVP	57.31	2.65	

### Questionnaires

#### Van der Laan’s technology acceptance score

The results of van der Laan’s technology acceptance score for the VVP with the surgeon and novice groups are listed in Table 2. In both groups, all the scale and item scores were above zero, which indicates a positive bias on the VVP in terms of its acceptance as a neoconceptual technology. The mean scale difference between the novice participants and surgeons was 0.36 in the usefulness scale and 0.56 in the satisfying scale. In terms of attributes, the “useful” attribute (in the usefulness scale) and “desirable” attribute (in the satisfying scale) received the highest scores in both groups.

**Table 2:** Results of van der Laan’s technology acceptance score

Attribute		Novice (*n* = 30)				Surgeon (*n* = 23)			
Scale	Item	Scale score		Item score		Scale score		Item score	
		Mean	SD	Mean	SD	Mean	SD	Mean	SD
Usefulness	Useful	1.23	0.82	1.63	0.49	0.87	0.84	1.04	0.98
	Good			1.00	0.87			0.87	0.69
	Effective			1.33	0.80			0.78	1.00
	Assisting			1.30	0.88			0.74	0.81
	Raising alertness			0.87	1.07			0.91	0.73
Satisfying	Pleasant	1.07	0.82	1.07	0.74	0.51	0.89	0.35	0.98
	Nice			1.03	0.89			0.61	0.84
	Likable			1.00	0.79			0.35	0.93
	Desirable			1.17	0.87			0.74	0.81

The attributes including scale and item range are between − 2 and + 2 based on a five-point Likert scale, with higher values implying a positive tendency

#### System usability scale

**Fig. 6:**
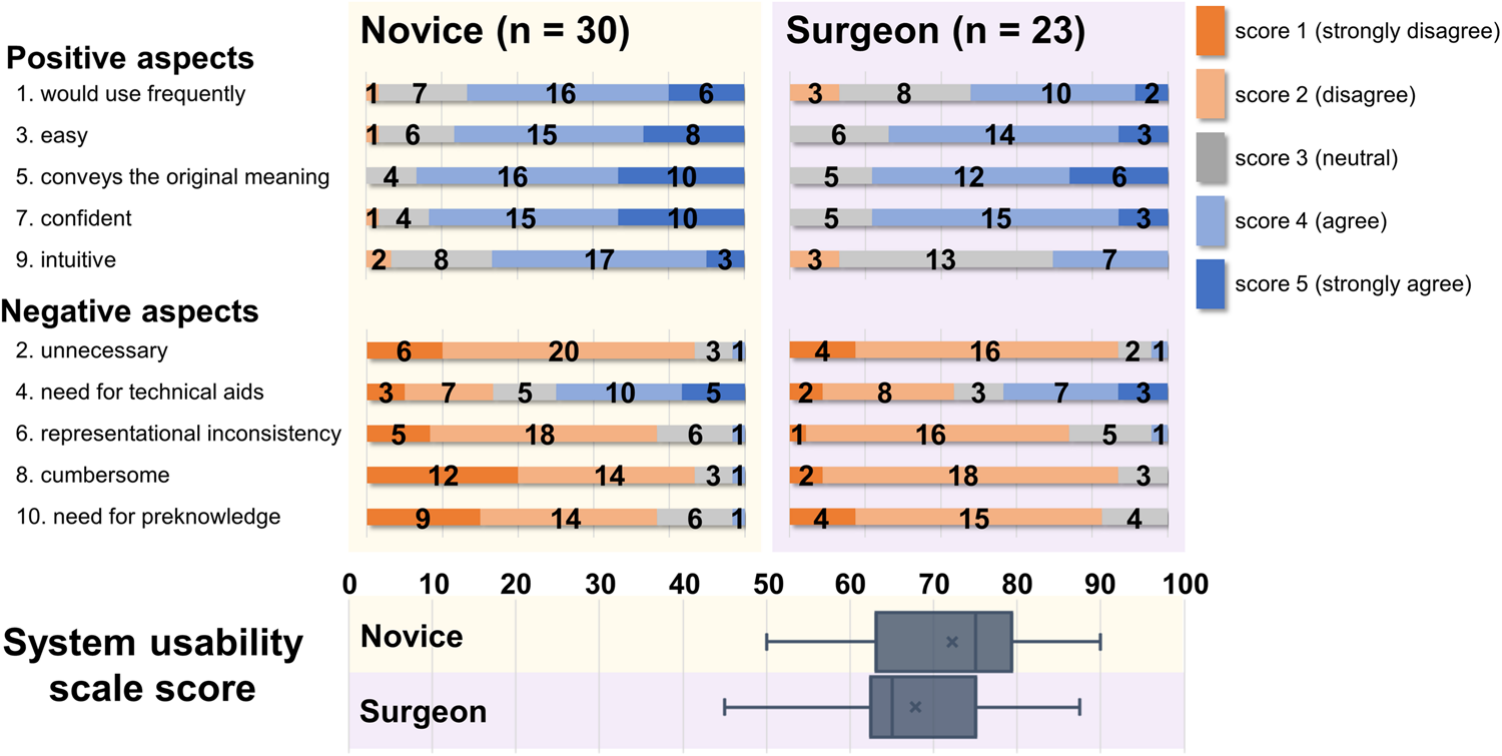
System usability scale. Scores are assigned based on five-point Likert scale

The questions of the SUS assessment for the VVP are categorized as positive (Question Nos. 1, 3, 5, 7, and 9) and negative (Question Nos. 2, 4, 6, 8, and 10) attributes. As demonstrated in Fig. 6, both groups evaluated the VVP with higher scores in terms of their positive attributes and lower scores in terms of their negative attributes. The comprehensive SUS score that reflects all responses to the questions estimated using the scoring formula was 70.33, which was adjacent to a “GOOD” adjective rating in the grade ranking of the SUS score [49].

#### NASA TLX

The NASA TLX was used in this study to compare the integrated workload when performing the RTT with each vision system. The results of the NASA TLX are summarized in Table 3. The global score was calculated based on the average values of the mental demand, physical demand, temporal demand, performance, effort, and frustration. Except for the physical demand in the novice group, there were no significant differences in the other indicators including the global score.

**Table 3:** Detailed numerical results of the comparative assessment

Criteria	Novice (*n* = 30)					Surgeon (*n* = 23)				
	SV		VVP		*p* value	SV		VVP		*p* value
	Mean	SD	Mean	SD		Mean	SD	Mean	SD	
Mental demand	4.53	2.19	4.77	2.16	0.680	4.30	2.01	4.43	2.11	0.824
Physical demand	5.10	1.99	3.90	1.40	0.009	4.61	1.95	4.39	2.04	0.592*
Temporal demand	4.40	2.11	4.23	2.08	0.759	4.65	1.67	4.43	1.67	0.603
Performance	6.93	2.21	7.07	2.03	0.081	5.96	1.99	5.87	1.77	0.737
Effort	6.00	1.78	5.73	2.03	0.591	5.52	1.97	5.87	1.69	0.603
Frustration	3.37	2.11	2.97	1.35	0.385	4.22	1.88	4.64	2.02	0.481
Global score	5.06	2.07	4.78	1.84	0.355	4.88	1.91	4.94	1.88	0.817

The indicators range between + 1 and + 10 based on a 10-point Likert scale, which means that the closer the score is to + 1, the lower is the task load, and the closer the score is to + 10, the higher is the task load. The *p* value is calculated by the independent sample *t* test and the *p* value (with the attached asterisk (*)) obtained by the Mann–Whitney *U* test

#### Comparative assessment

**Fig. 7:**
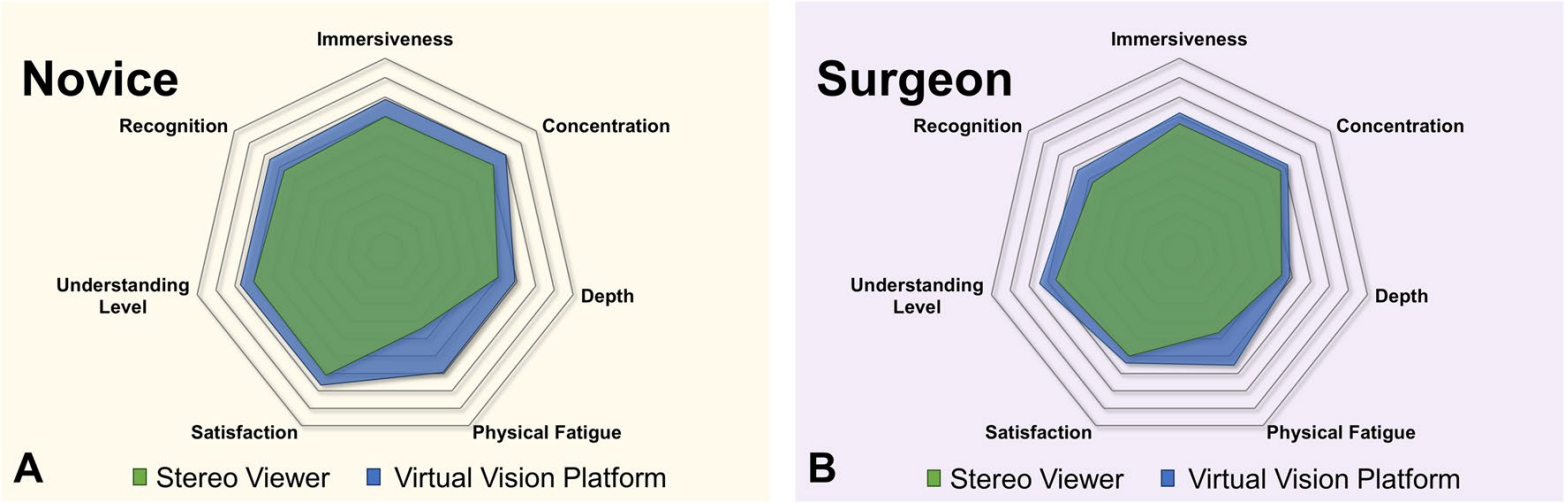
Comparison of SV and VVP. **A** Novice group. **B** Surgeon group

The comprehensive tendency of the respective indicators between the vision systems are shown in Fig. 7, and detailed numerical values are presented in Table 4. In both groups, there were higher scores toward the VVP in all indicators. In the case of concentration in the novice group and physical fatigue in both groups, statistical differences were observed between the SV and VVP.

**Table 4:** Numerical results of the comparative assessments

Criteria	Novice (*n* = 30)					Surgeon (*n* = 23)				
	SV		VVP		*p* value	SV		VVP		*p* value
	Mean	SD	Mean	SD		Mean	SD	Mean	SD	
Immersiveness	6.97	2.01	7.87	1.28	0.044	6.61	1.62	7.17	1.59	0.178*
Concentration	7.17	1.74	8.00	1.20	0.035	6.70	1.43	7.17	1.37	0.195*
Depth	6.00	2.45	6.90	2.07	0.130	5.43	2.04	5.87	1.77	0.469
Physical fatigue	4.40	1.90	6.93	2.10	0.000	4.65	1.50	6.52	1.70	0.001
Satisfaction	7.10	1.92	7.67	1.49	0.207	6.00	1.98	6.39	1.47	0.563*
Understanding level	7.00	1.72	7.70	1.66	0.115	6.57	1.80	7.43	1.24	0.090*
Recognition	6.70	2.20	7.63	1.54	0.062	5.74	1.89	6.74	1.74	0.050

The indicators range between + 1 and + 10 based on a 10-point Likert scale, which means that the closer the score is to + 1, the lower the task load is, and the closer the score is to + 10, the higher the task load. The general *p* value is calculated using the independent sample *t* test and the *p* value attached asterisk (*) obtained by the Mann–Whitney *U* test

#### Optimal arrangement of multiple medical displays

**Fig. 8:**
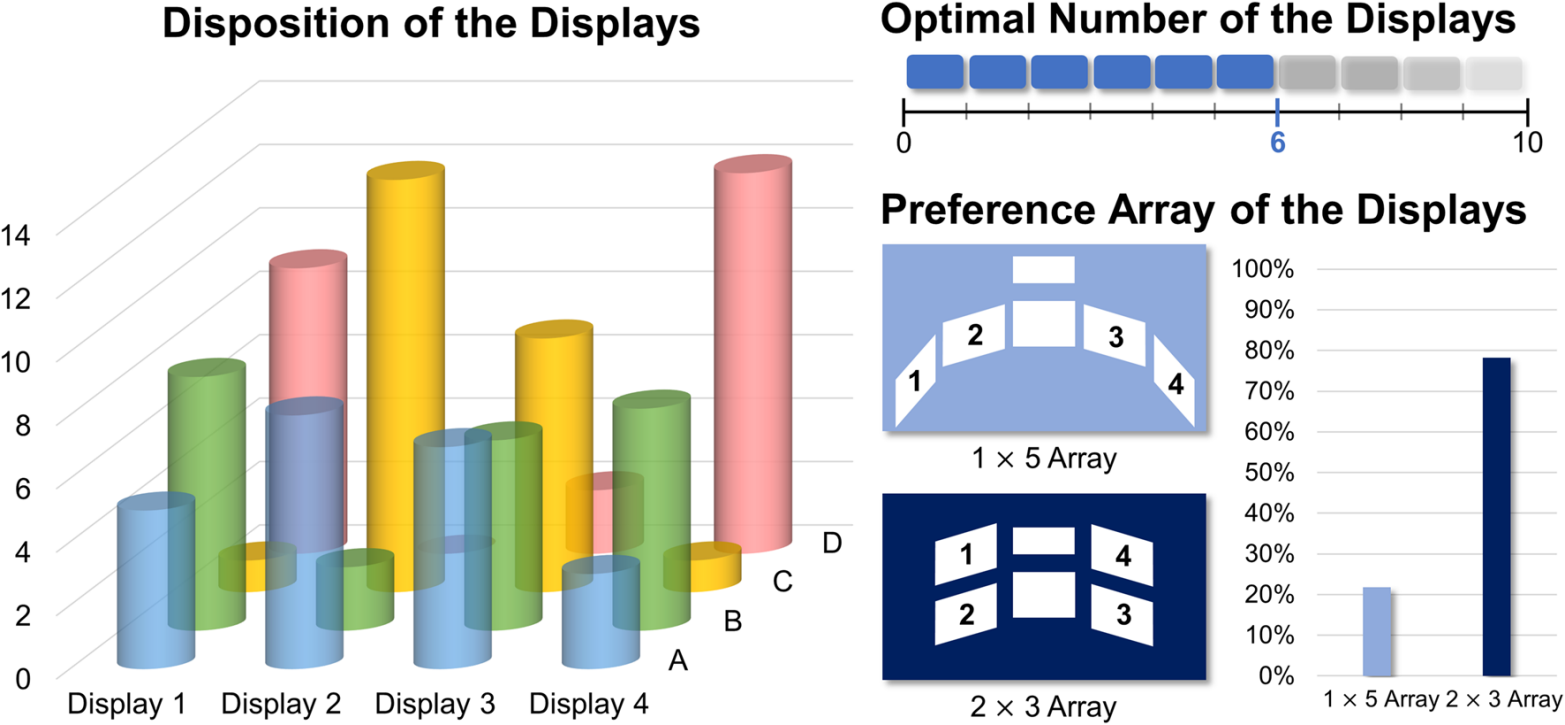
Questionnaire results regarding the arrangement of the displays. **A** Captured image. **B** Vital sign viewer. **C** Digital imaging communication in medicine (DICOM) viewer. **D** Electronic medical records (EMR)

The VVP can be customized by the arrangement of multiple medical displays according to the individual user’s preference. Therefore, a comprehensive survey about the most appropriate arrangement was conducted among the surgeon group members to reflect their expertise and knowledge about the surroundings of the operation room, as shown in Fig. 8. The survey shows the surgeons tended to prefer that the captured image (A) be placed on Display 2, the vital sign viewer (B) placed on Display 1, the DICOM viewer (C) on Display 2, and the EMR (D) placed on Display 4. The surgeons stated that the optimal number of displays was six on the median, including the surgery view and timer in the middle part of the VR space, and preferred a 2 × 3 array (78.26%) rather than a 1 × 5 array (21.74%).

## Discussion

In the present study, the usability of the VVP, which provides various medical information through the binocular screens of an HMD, was investigated in terms of ergonomics. The user evaluation was implemented by utilizing the dVRK. Participants with different levels of familiarity with the RAS platform were included to identify a global solution for the VVP. All participants executed the RTT in conjunction with two scenarios—DVC and VSC—and responded to the questionnaire survey questions based on their experience when performing the RTT. The scenarios were designed based on the situations that can occur in the operating room to investigate the diverse interactions with other medical information needed for surgery. Therefore, the type and number of medical information on the left and right sides of the VVP were identical for all participants to analyze the interactive tendency under the same conditions. The results of the user evaluation are analyzed below.

In the RTT, there were no significant differences in the success rate, time required to transfer a ring, and time required to execute the scenarios with the use of the SV and VVP. This indicates that the performance using the VVP does not differ significantly from that when the SV is used. The CVA results statistically demonstrated a postural advantage for the VVP over SV with the value being 16.35° higher on average in the VVP than in the SV (*p* < 0.05). The reason the standard deviation of the CVA was higher in the VVP can be attributed to the increased number of DOFs in the head. Additionally, this finding is supported by the indicators relevant to fatigue in the questionnaire responses. In the DVC and VSC scenarios added to evaluate whether a surgeon could effectively identify the information needed during the surgery, the times required for performing the respective scenarios in the SV and VVP cases were not significantly different. This finding can support the assertion that surgeons can manage the acquisition and processing of the medical information for the patient, regardless of the vision system. It is expected to have a meaningful difference in an operating room, because the distance between the SV and other monitors is longer than the experimental environment designed in the present study. Due to the procedural omission of repeatedly taking on and off the HMD to check the data not provided in the SV, the surgeon can operate and assess important patient data simultaneously, leading to a continuous surgical flow.

Various questionnaires, including van der Laan’s technology acceptance score, SUS, NASA TLX, and comparative assessment, were implemented after completing the RTT to reflect the opinion based on the experience with the respective vision system. Van der Laan’s technology acceptance score and SUS were executed only for the VVP case; by contrast, NASA TLX and comparative assessment were conducted to compare the ergonomic properties of the SV and VVP. As listed in Table 2, the technology acceptance score for the VVP was convincing for both surgeons and novices as all scores were higher than the middle score point. The SUS results also showed a positive tendency about the VVP in all questions in both groups. Furthermore, the comprehensive SUS score was 70.33, which supports the system usability of the VVP in terms of ergonomics [49, 50].

In the NASA TLX and comparative assessments, the overall results for the VVP were positive. Among the results, the scores for the physical demand in the novice group in the NASA TLX and physical fatigue in the comparative assessment in both groups were statistically significant. This supports the interpretation of the postural advantage based on the CVA. In cases of physical and temporal demands, the scores were lower in VVP than in the SV in both groups, indicating that the load caused by inconvenient posture can be alleviated by the VVP. Moreover, the tendencies in performance, effort, and frustration were different between the surgeon and novice groups. These differences were caused by the differences in familiarity with the SV according to the usage period of the surgeons. In the case of mental demand, the score using the VVP was higher than the SV owing to the hardware limitations of the HMD, such as restricted resolution and sense of weight; however, these scores can be improved if the HMD is customized to the needed specifications for medical purposes. Considering the alleviated physical load and potential for further study, the VVP can be used in a novel vision system.

To investigate the arrangement of multiple medical displays, a survey was conducted to determine the standardized composition of medical information in the VR-based world. While the results showed that the median optimal number of the displays was six and the preferred arrangement was a 2 × 3 array, there was no common standardized formation in the display disposition. Based on the interviews with the surgeons, this is attributed to the fact that the necessity of the given information was different for each department as well as for each surgeon’s preference in the same department; however, it is evident that they wanted to locate the important information in Displays 2 or 3. Based on the tendency in the present study, the VVP must be developed to reflect and customize individual preferences, which is expected to create new prospects and opportunities in the VR-based medical field. Furthermore, the customized VVP can be multilaterally analyzed using a variety of parameters from the globally reliable questionnaires, such as after-scenario questionnaire (ASQ), computer system usability questionnaire (CSUQ), and questionnaire for user interface satisfaction (QUIS) [57]. Considering the novel and growing interest in leveraging visual feedback, such as the mechanochromic surgical tools in the MIS and RAS, more efficiently for users, the VVP introduces innovations that enable the provision of multiple medical information within a virtual space, tailored to specific user preferences [58]. It plays a crucial role in contributing to the advancement toward the preclinical and clinical fields, aligning with the vision-based progressive development of future-oriented surgical robotics.

## Conclusions

According to the global usage of the RAS platform, such as the dVSS, the ergonomic improvement of a surgical robot for the surgeon has become a necessity in the medical field. By adopting an HMD as a new vision system to alleviate chronic fatigue, the additional issue associated with the repeated removal and donning of HMD to assess the generation of external information, which induces discontinuous surgical flow, must be addressed. To this end, a VVP that simultaneously provides multiple types of medical information in the VR-based world was proposed and investigated in the present study. Consequently, surgeons and novices demonstrated similar task performance between the given vision systems, with a more positive tendency toward the VVP based on questionnaire responses and postural advantages. Considering the improvements investigated in the present study, the VVP can become a candidate as a neoconceptual vision system for the next-generation RAS platform.

### Supplementary Information

Below is the link to the electronic supplementary material.

Supplementary file1 (PDF 537 KB)
