# Supplementary material for: A multimodal virtual vision platform as a next-generation vision system for a surgical robot
Source: Med Biol Eng Comput. 2024 Feb 2;62(5):1535–48. doi: 10.1007/s11517-024-03030-1 (PMC11021270; doi:10.1007/s11517-024-03030-1)
Supplement: Supplementary file 1 — Supplementary file1 (PDF 537 KB) [file 11517_2024_3030_MOESM1_ESM.pdf]

# Supplementary Materials

**Table A** Detailed results of the ring transfer task when using the stereo viewer in the novice group

| No. | Indicator | Number of Ring | | | | | | | | | | | Success rate (%) | Time for transferring a ring | |
|---|---|---|---|---|---|---|---|---|---|---|---|---|---|---|---|
| | | 1 | 2 | 3 | 4 | 5 | 6 | 7 | 8 | 9 | 10 | 11 | | Mean | SD |
| 1 | Success / Fail | S | S | S | S | F | S | S | F | S | F | S | 72.73 | 27.26 | 12.21 |
| | Time | 26.56 | 22.62 | 22.20 | 56.73 | 23.86 | 24.35 | 26.30 | 58.05 | 18.70 | 13.46 | 20.59 | | | |
| 2 | Success / Fail | F | S | S | F | S | S | S | F | F | S | S | 63.64 | 53.11 | 9.90 |
| | Time | 45.27 | 69.44 | 57.27 | 16.69 | 46.72 | 46.37 | 44.05 | 34.81 | 37.67 | 50.06 | 64.62 | | | |
| 3 | Success / Fail | S | F | S | S | S | S | S | S | S | S | S | 90.91 | 64.21 | 24.89 |
| | Time | 63.85 | 23.21 | 84.23 | 84.17 | 69.95 | 102.82 | 39.91 | 61.00 | 77.97 | 29.64 | 28.60 | | | |
| 4 | Success / Fail | S | F | S | F | S | S | S | S | S | F | S | 72.73 | 50.42 | 18.11 |
| | Time | 52.32 | 7.10 | 26.90 | 26.30 | 39.49 | 75.20 | 56.74 | 29.43 | 49.93 | 18.83 | 73.31 | | | |
| 5 | Success / Fail | S | S | S | S | S | S | S | S | S | S | S | 100.00 | 49.03 | 12.57 |
| | Time | 40.31 | 52.50 | 37.58 | 54.13 | 22.67 | 52.94 | 62.44 | 51.35 | 68.84 | 43.47 | 53.11 | | | |
| 6 | Success / Fail | S | S | S | F | S | S | S | S | S | S | S | 90.91 | 25.89 | 9.35 |
| | Time | 32.84 | 23.71 | 26.08 | 25.00 | 35.19 | 31.15 | 14.79 | 11.90 | 20.96 | 41.65 | 20.62 | | | |
| 7 | Success / Fail | S | S | S | S | S | S | S | S | S | S | F | 90.91 | 42.72 | 20.37 |
| | Time | 37.25 | 66.29 | 86.26 | 37.23 | 32.40 | 48.25 | 46.02 | 24.66 | 30.76 | 18.10 | 14.62 | | | |
| 8 | Success / Fail | S | S | S | S | S | S | S | S | S | F | F | 81.82 | 69.21 | 77.17 |
| | Time | 34.56 | 35.50 | 44.50 | 48.90 | 64.42 | 273.27 | 32.21 | 39.60 | 49.94 | 27.98 | 18.23 | | | |
| 9 | Success / Fail | S | S | S | S | S | S | S | S | S | S | S | 100.00 | 29.67 | 12.58 |
| | Time | 21.70 | 22.09 | 23.38 | 28.19 | 27.95 | 39.87 | 13.61 | 21.48 | 26.82 | 57.72 | 43.57 | | | |

| | | | | | | | | | | | | | | | |
|---|---|---|---|---|---|---|---|---|---|---|---|---|---|---|---|
| 10 | Success / Fail | S | S | S | F | S | F | S | S | S | S | S | 81.82 | 25.25 | 8.04 |
| | Time | 35.28 | 20.84 | 25.07 | 17.30 | 32.46 | 9.54 | 16.47 | 18.05 | 19.91 | 20.94 | 38.24 | | | |
| 11 | Success / Fail | S | S | S | S | S | S | S | S | S | S | S | 100.00 | 20.82 | 4.87 |
| | Time | 18.05 | 31.60 | 19.59 | 16.04 | 19.69 | 15.34 | 17.87 | 18.31 | 22.15 | 24.14 | 26.28 | | | |
| 12 | Success / Fail | S | S | S | S | S | S | S | S | S | S | S | 100.00 | 25.76 | 9.06 |
| | Time | 11.99 | 20.74 | 17.90 | 45.94 | 23.91 | 22.43 | 26.42 | 22.46 | 27.15 | 28.67 | 35.79 | | | |
| 13 | Success / Fail | S | F | F | S | F | S | S | S | S | S | S | 72.73 | 24.58 | 11.16 |
| | Time | 13.83 | 7.85 | 11.52 | 14.74 | 5.91 | 35.82 | 20.14 | 26.31 | 45.93 | 17.59 | 22.24 | | | |
| 14 | Success / Fail | S | S | S | S | S | S | S | S | S | S | S | 100.00 | 31.04 | 7.03 |
| | Time | 38.73 | 21.21 | 28.02 | 30.18 | 25.66 | 31.38 | 43.11 | 37.59 | 20.72 | 31.67 | 33.14 | | | |
| 15 | Success / Fail | S | S | S | S | S | S | S | S | F | S | S | 90.91 | 25.56 | 11.92 |
| | Time | 40.28 | 24.07 | 27.71 | 14.49 | 23.32 | 20.04 | 51.50 | 17.45 | 11.55 | 23.66 | 13.07 | | | |
| 16 | Success / Fail | S | S | S | S | S | S | F | S | S | F | S | 81.82 | 49.81 | 17.44 |
| | Time | 48.50 | 38.22 | 59.96 | 54.61 | 79.32 | 43.25 | 98.01 | 67.56 | 32.26 | 29.75 | 24.61 | | | |
| 17 | Success / Fail | S | S | S | S | F | S | S | S | S | S | S | 90.91 | 30.98 | 11.04 |
| | Time | 25.27 | 30.26 | 23.01 | 22.75 | 20.74 | 24.58 | 25.73 | 56.49 | 34.66 | 23.85 | 43.17 | | | |
| 18 | Success / Fail | S | S | S | S | S | F | S | S | S | S | S | 90.91 | 24.11 | 3.21 |
| | Time | 23.03 | 20.42 | 19.64 | 25.90 | 24.77 | 10.76 | 22.48 | 25.09 | 25.51 | 31.02 | 23.23 | | | |
| 19 | Success / Fail | F | S | S | S | S | S | S | S | F | S | S | 81.82 | 40.05 | 12.36 |
| | Time | 20.35 | 32.15 | 63.11 | 40.00 | 48.85 | 16.82 | 37.89 | 40.78 | 39.07 | 38.78 | 42.09 | | | |
| 20 | Success / Fail | S | S | S | S | F | S | S | S | F | S | S | 81.82 | 30.19 | 6.63 |
| | Time | 23.08 | 23.29 | 39.39 | 35.20 | 31.98 | 34.58 | 25.52 | 26.37 | 22.45 | 25.79 | 38.50 | | | |
| 21 | Success / Fail | S | S | F | S | S | S | S | S | F | S | S | 81.82 | 21.69 | 5.35 |
| | Time | 30.78 | 21.42 | 5.45 | 15.06 | 16.96 | 25.86 | 16.58 | 18.48 | 30.44 | 26.40 | 23.68 | | | |
| 22 | Success / Fail | F | S | S | S | S | S | S | S | S | S | S | 90.91 | 27.27 | 8.74 |

| | | | | | | | | | | | | | | | |
|---|---|---|---|---|---|---|---|---|---|---|---|---|---|---|---|
| | Time | 19.58 | 34.18 | 21.60 | 24.29 | 22.96 | 48.84 | 20.84 | 30.59 | 20.79 | 23.02 | 25.60 | | | |
| 23 | Success / Fail | S | S | S | S | S | S | S | S | S | S | S | 100.00 | 31.35 | 7.64 |
| | Time | 47.36 | 30.82 | 29.15 | 27.42 | 37.66 | 36.24 | 23.27 | 26.32 | 37.81 | 21.66 | 27.18 | | | |
| 24 | Success / Fail | S | S | S | S | F | S | S | S | S | S | S | 90.91 | 25.05 | 10.58 |
| | Time | 22.59 | 27.25 | 11.96 | 49.95 | 9.69 | 17.78 | 21.42 | 8..72 | 28.98 | 23.74 | 21.78 | | | |
| 25 | Success / Fail | S | S | F | F | S | S | S | S | S | S | S | 81.82 | 62.99 | 26.13 |
| | Time | 34.63 | 43.81 | 23.81 | 37.01 | 78.46 | 46.22 | 49.23 | 84.98 | 117.51 | 52.45 | 59.60 | | | |
| 26 | Success / Fail | S | S | S | S | S | S | S | S | S | S | S | 100.00 | 31.35 | 12.57 |
| | Time | 34.57 | 34.18 | 50.88 | 57.91 | 27.50 | 22.00 | 17.85 | 22.07 | 28.04 | 27.07 | 22.74 | | | |
| 27 | Success / Fail | F | S | S | S | S | F | S | S | F | S | S | 72.73 | 25.19 | 7.65 |
| | Time | 17.93 | 20.54 | 24.45 | 36.00 | 19.37 | 40.22 | 19.61 | 25.87 | 14.76 | 37.62 | 18.06 | | | |
| 28 | Success / Fail | S | S | S | S | S | S | S | S | S | S | S | 100.00 | 29.63 | 12.16 |
| | Time | 30.62 | 16.76 | 19.23 | 22.64 | 36.39 | 23.17 | 26.75 | 26.10 | 59.93 | 39.83 | 24.56 | | | |
| 29 | Success / Fail | F | S | F | S | S | S | S | S | S | S | S | 81.82 | 43.69 | 26.25 |
| | Time | 22.81 | 31.50 | 15.03 | 27.14 | 26.22 | 44.18 | 54.65 | 43.63 | 27.51 | 108.57 | 29.77 | | | |
| 30 | Success / Fail | S | S | S | S | S | S | S | S | S | S | S | 100.00 | 30.00 | 13.05 |
| | Time | 25.70 | 57.38 | 24.85 | 52.66 | 22.67 | 26.83 | 17.55 | 34.01 | 23.76 | 21.45 | 23.14 | | | |

**Table B** Detailed results of the ring transfer task when using the virtual vision platform in the novice group

| No. | Indicator | Number of Ring | | | | | | | | | | | Success rate (%) | Time for transferring a ring | |
|---|---|---|---|---|---|---|---|---|---|---|---|---|---|---|---|
| | | 1 | 2 | 3 | 4 | 5 | 6 | 7 | 8 | 9 | 10 | 11 | | Mean | SD |
| 1 | Success / Fail | S | F | S | S | S | F | S | S | S | S | S | 81.82 | 36.58 | 17.92 |
| | Time | 59.68 | 25.80 | 14.91 | 54.30 | 19.11 | 34.67 | 20.61 | 60.32 | 41.34 | 27.82 | 31.13 | | | |
| 2 | Success / Fail | S | S | S | S | S | S | S | S | F | F | F | 72.73 | 60.25 | 17.35 |
| | Time | 42.36 | 70.53 | 71.78 | 41.68 | 81.99 | 66.56 | 70.90 | 36.19 | 25.99 | 12.46 | 71.08 | | | |
| 3 | Success / Fail | S | S | F | S | S | F | S | S | F | S | S | 72.73 | 44.27 | 12.15 |
| | Time | 34.09 | 36.19 | 31.20 | 44.68 | 28.56 | 49.00 | 61.48 | 39.97 | 27.26 | 61.27 | 47.93 | | | |
| 4 | Success / Fail | S | F | S | S | S | S | S | S | S | S | S | 90.91 | 41.93 | 19.78 |
| | Time | 44.66 | 55.03 | 33.39 | 41.80 | 22.48 | 32.50 | 32.07 | 40.53 | 51.77 | 27.49 | 92.62 | | | |
| 5 | Success / Fail | S | S | S | S | S | S | S | S | F | S | S | 90.91 | 60.79 | 19.13 |
| | Time | 39.70 | 69.33 | 34.79 | 45.94 | 59.88 | 51.15 | 99.16 | 77.09 | 90.50 | 66.70 | 64.19 | | | |
| 6 | Success / Fail | S | S | F | S | S | S | S | S | S | S | S | 90.91 | 38.55 | 17.24 |
| | Time | 31.54 | 24.48 | 31.95 | 23.50 | 44.76 | 19.92 | 31.10 | 64.74 | 57.24 | 25.85 | 62.32 | | | |
| 7 | Success / Fail | S | F | S | S | S | S | F | S | S | S | S | 81.82 | 41.50 | 6.16 |
| | Time | 35.98 | 53.55 | 46.25 | 41.78 | 46.91 | 29.33 | 17.50 | 49.15 | 44.34 | 39.24 | 40.48 | | | |
| 8 | Success / Fail | F | S | S | S | S | S | S | S | F | S | S | 81.82 | 34.40 | 13.51 |
| | Time | 69.44 | 29.63 | 27.15 | 64.37 | 31.35 | 29.29 | 16.43 | 41.29 | 7.52 | 28.75 | 41.34 | | | |
| 9 | Success / Fail | S | S | S | S | S | S | S | S | S | F | S | 90.91 | 28.77 | 6.44 |
| | Time | 23.09 | 28.49 | 25.35 | 41.58 | 23.66 | 32.24 | 22.75 | 22.65 | 34.04 | 21.30 | 33.82 | | | |
| 10 | Success / Fail | S | S | S | S | S | S | F | F | S | S | F | 72.73 | 36.44 | 7.47 |
| | Time | 28.34 | 25.23 | 43.50 | 35.90 | 44.04 | 35.55 | 17.65 | 74.22 | 33.43 | 45.50 | 35.12 | | | |
| 11 | Success / Fail | S | S | S | S | S | S | S | S | S | S | S | 100.00 | 28.76 | 12.73 |

| | | | | | | | | | | | | | | | |
|---|---|---|---|---|---|---|---|---|---|---|---|---|---|---|---|
| | Time | 24.93 | 15.50 | 37.40 | 27.12 | 15.78 | 40.60 | 18.52 | 18.50 | 57.21 | 33.58 | 27.24 | | | |
| 12 | Success / Fail | S | F | S | S | S | F | S | S | S | S | S | 81.82 | 39.21 | 13.61 |
| | Time | 24.96 | 28.51 | 30.41 | 37.30 | 25.47 | 31.91 | 44.20 | 60.70 | 35.01 | 61.02 | 33.83 | | | |
| 13 | Success / Fail | S | S | S | F | S | S | F | S | S | S | S | 81.82 | 32.09 | 13.69 |
| | Time | 34.65 | 64.99 | 33.84 | 19.65 | 19.52 | 26.13 | 36.93 | 33.19 | 32.56 | 19.98 | 23.92 | | | |
| 14 | Success / Fail | S | F | F | S | S | S | S | S | S | S | S | 81.82 | 28.25 | 8.01 |
| | Time | 16.97 | 25.62 | 18.10 | 37.81 | 16.33 | 29.93 | 26.30 | 34.48 | 25.45 | 38.17 | 28.80 | | | |
| 15 | Success / Fail | S | S | S | S | S | S | S | S | S | F | S | 90.91 | 33.88 | 14.46 |
| | Time | 16.84 | 17.47 | 33.22 | 37.32 | 45.10 | 29.80 | 29.05 | 31.13 | 31.34 | 14.10 | 67.48 | | | |
| 16 | Success / Fail | S | S | S | S | S | S | S | S | S | F | S | 90.91 | 58.42 | 9.45 |
| | Time | 52.73 | 46.79 | 50.67 | 55.85 | 74.59 | 61.93 | 67.34 | 66.54 | 61.22 | 74.78 | 46.49 | | | |
| 17 | Success / Fail | F | S | S | S | F | S | S | S | S | S | S | 81.82 | 33.01 | 7.81 |
| | Time | 45.29 | 33.11 | 29.67 | 26.25 | 35.98 | 39.79 | 43.36 | 25.39 | 27.01 | 45.29 | 27.20 | | | |
| 18 | Success / Fail | S | S | S | S | S | S | S | S | S | S | F | 90.91 | 32.89 | 6.68 |
| | Time | 38.72 | 36.75 | 27.24 | 40.18 | 27.38 | 35.53 | 19.64 | 36.67 | 28.87 | 37.87 | 13.50 | | | |
| 19 | Success / Fail | S | F | S | S | S | S | S | S | S | S | S | 90.91 | 32.15 | 9.00 |
| | Time | 38.28 | 15.57 | 34.38 | 33.82 | 45.18 | 20.50 | 15.87 | 32.35 | 27.52 | 41.71 | 31.89 | | | |
| 20 | Success / Fail | S | S | S | S | S | S | S | S | F | S | F | 81.82 | 53.05 | 13.04 |
| | Time | 54.03 | 50.80 | 47.42 | 67.70 | 43.91 | 76.42 | 33.44 | 45.08 | 84.62 | 58.62 | 14.74 | | | |
| 21 | Success / Fail | S | S | S | S | S | F | F | S | S | S | S | 81.82 | 28.69 | 6.13 |
| | Time | 28.30 | 25.96 | 25.65 | 37.82 | 26.81 | 6.14 | 25.19 | 30.38 | 22.22 | 22.03 | 39.05 | | | |
| 22 | Success / Fail | F | S | S | S | S | S | F | S | F | S | S | 72.73 | 46.06 | 18.04 |
| | Time | 34.57 | 44.56 | 34.95 | 21.98 | 51.24 | 33.32 | 43.68 | 43.78 | 19.05 | 57.18 | 81.48 | | | |
| 23 | Success / Fail | S | S | S | S | S | S | S | S | S | S | S | 100.00 | 32.65 | 19.17 |
| | Time | 31.10 | 39.57 | 31.43 | 21.36 | 32.41 | 20.83 | 19.79 | 25.98 | 27.79 | 87.41 | 21.50 | | | |

| | | | | | | | | | | | | | | | |
|---|---|---|---|---|---|---|---|---|---|---|---|---|---|---|---|
| 24 | Success / Fail | S | S | S | S | S | S | S | S | S | S | S | 100.00 | 27.20 | 10.92 |
| | Time | 42.00 | 15.74 | 20.55 | 30.03 | 49.61 | 28.35 | 19.54 | 19.42 | 23.25 | 33.82 | 16.85 | | | |
| 25 | Success / Fail | S | S | S | S | S | S | S | S | S | S | S | 100.00 | 77.81 | 21.26 |
| | Time | 52.35 | 56.38 | 111.65 | 64.28 | 65.75 | 88.26 | 92.27 | 68.04 | 69.06 | 72.35 | 115.48 | | | |
| 26 | Success / Fail | S | S | S | S | S | S | S | S | S | S | S | 100.00 | 43.96 | 12.01 |
| | Time | 65.12 | 48.47 | 33.97 | 30.57 | 47.03 | 37.7 | 64.75 | 43.07 | 33.38 | 45.47 | 33.98 | | | |
| 27 | Success / Fail | F | S | S | S | S | S | S | S | S | S | S | 90.91 | 19.96 | 8.77 |
| | Time | 5.67 | 14.93 | 13.67 | 22.3 | 36.71 | 34.69 | 17.05 | 18.63 | 14.87 | 13.73 | 12.98 | | | |
| 28 | Success / Fail | S | S | S | S | S | S | S | S | S | S | S | 100.00 | 33.78 | 8.65 |
| | Time | 23.74 | 25.18 | 25.07 | 41.49 | 51.3 | 41.26 | 29.92 | 27.86 | 32.29 | 34.99 | 38.46 | | | |
| 29 | Success / Fail | S | S | S | S | S | F | S | S | S | S | S | 90.91 | 43.64 | 16.15 |
| | Time | 59.84 | 25.96 | 58.31 | 28.32 | 31.49 | 10.98 | 75.33 | 31.61 | 40.4 | 45.14 | 40.03 | | | |
| 30 | Success / Fail | S | S | F | S | S | S | S | S | S | S | S | 90.91 | 49.20 | 18.16 |
| | Time | 43.01 | 44.41 | 21.02 | 47.29 | 35.13 | 38.91 | 98.17 | 51.12 | 35.50 | 51.58 | 46.83 | | | |

**Table C** Detailed results of the ring transfer task when using the stereo viwer in the surgeon group

| No. | Indicator | Number of Ring | | | | | | | | | | | Success rate (%) | Time for transferring a ring | |
|---|---|---|---|---|---|---|---|---|---|---|---|---|---|---|---|
| | | 1 | 2 | 3 | 4 | 5 | 6 | 7 | 8 | 9 | 10 | 11 | | Mean | SD |
| 1 | Success / Fail | S | S | S | S | S | S | F | S | S | S | F | 81.82 | 23.21 | 7.21 |
| | Time | 26.22 | 21.92 | 17.69 | 22.41 | 25.46 | 40.06 | 16.06 | 20.94 | 15.43 | 18.78 | 6.97 | | | |
| 2 | Success / Fail | S | S | S | S | S | S | S | S | S | S | S | 100.00 | 23.93 | 13.82 |
| | Time | 14.89 | 26.18 | 13.24 | 61.13 | 21.87 | 19.70 | 16.53 | 12.26 | 17.94 | 31.80 | 27.74 | | | |
| 3 | Success / Fail | S | S | S | S | S | S | S | S | S | F | S | 90.91 | 31.05 | 11.57 |
| | Time | 32.13 | 33.46 | 25.32 | 24.66 | 25.29 | 24.68 | 17.78 | 33.75 | 60.43 | 12.14 | 33.02 | | | |
| 4 | Success / Fail | S | S | S | F | F | S | S | F | S | S | S | 72.73 | 18.82 | 4.07 |
| | Time | 20.40 | 19.48 | 14.53 | 8.59 | 20.22 | 26.93 | 20.09 | 16.46 | 16.91 | 13.98 | 18.23 | | | |
| 5 | Success / Fail | S | F | F | S | S | S | S | S | S | S | S | 81.82 | 27.66 | 14.08 |
| | Time | 62.99 | 19.07 | 21.81 | 24.34 | 20.49 | 20.87 | 16.80 | 29.91 | 23.41 | 18.93 | 31.22 | | | |
| 6 | Success / Fail | S | F | S | S | S | S | S | S | S | S | S | 90.91 | 27.32 | 7.12 |
| | Time | 36.74 | 30.59 | 28.83 | 31.95 | 27.94 | 24.59 | 21.79 | 20.02 | 21.52 | 40.03 | 19.74 | | | |
| 7 | Success / Fail | S | S | S | S | S | S | S | F | F | S | S | 81.82 | 40.49 | 24.40 |
| | Time | 104.49 | 34.59 | 30.04 | 30.59 | 37.94 | 28.90 | 40.65 | 12.81 | 18.68 | 28.22 | 29.00 | | | |
| 8 | Success / Fail | S | S | S | S | S | S | S | S | F | S | S | 90.91 | 25.88 | 4.42 |
| | Time | 23.18 | 36.77 | 20.40 | 27.50 | 25.30 | 24.42 | 23.75 | 24.13 | 22.74 | 24.96 | 28.41 | | | |
| 9 | Success / Fail | F | F | S | S | F | F | S | S | S | F | F | 45.45 | 34.68 | 10.46 |
| | Time | 18.73 | 31.09 | 47.89 | 28.32 | 14.57 | 36.67 | 33.06 | 21.90 | 42.23 | 20.94 | 34.35 | | | |
| 10 | Success / Fail | S | S | S | S | S | S | S | S | S | S | S | 100.00 | 18.75 | 3.12 |
| | Time | 20.95 | 18.52 | 15.91 | 17.10 | 19.31 | 23.19 | 14.97 | 23.67 | 16.56 | 20.97 | 15.05 | | | |
| 11 | Success / Fail | S | S | S | S | S | S | S | F | S | S | S | 90.91 | 20.27 | 4.90 |

| | | | | | | | | | | | | | | | |
|---|---|---|---|---|---|---|---|---|---|---|---|---|---|---|---|
| | Time | 16.91 | 18.69 | 27.16 | 16.64 | 17.87 | 19.98 | 14.14 | 8.51 | 30.16 | 21.04 | 20.09 | | | |
| 12 | Success / Fail | S | S | S | S | S | S | S | S | S | S | S | 100.00 | 21.41 | 3.95 |
| | Time | 24.93 | 27.41 | 19.06 | 18.60 | 25.67 | 16.59 | 24.01 | 19.94 | 15.59 | 19.68 | 24.07 | | | |
| 13 | Success / Fail | S | S | S | S | S | S | S | S | S | S | S | 100.00 | 21.27 | 4.66 |
| | Time | 22.98 | 29.61 | 13.46 | 17.97 | 27.36 | 17.31 | 22.70 | 22.70 | 20.43 | 17.46 | 21.97 | | | |
| 14 | Success / Fail | F | F | S | S | S | S | S | S | F | S | S | 72.73 | 46.86 | 26.32 |
| | Time | 43.59 | 31.07 | 58.10 | 103.29 | 41.69 | 22.35 | 56.26 | 28.79 | 35.12 | 26.35 | 38.07 | | | |
| 15 | Success / Fail | S | S | S | F | S | S | S | S | S | S | S | 90.91 | 24.45 | 8.49 |
| | Time | 19.49 | 34.92 | 13.17 | 10.87 | 23.89 | 40.44 | 26.62 | 13.37 | 23.87 | 22.99 | 25.72 | | | |
| 16 | Success / Fail | F | S | S | S | F | S | F | S | S | S | S | 72.73 | 18.32 | 3.97 |
| | Time | 16.49 | 14.18 | 11.81 | 20.86 | 14.48 | 19.50 | 19.10 | 20.67 | 17.98 | 24.34 | 17.18 | | | |
| 17 | Success / Fail | S | S | S | S | S | S | S | S | S | S | S | 100.00 | 36.48 | 15.25 |
| | Time | 43.93 | 32.02 | 27.04 | 44.76 | 75.86 | 35.51 | 34.04 | 18.07 | 34.30 | 23.30 | 32.48 | | | |
| 18 | Success / Fail | S | S | S | S | S | F | S | S | F | S | S | 81.82 | 32.85 | 16.82 |
| | Time | 55.90 | 26.88 | 23.77 | 26.34 | 15.46 | 12.17 | 17.63 | 54.94 | 1.70 | 21.45 | 53.31 | | | |
| 19 | Success / Fail | S | S | S | S | S | S | S | S | S | S | F | 90.91 | 21.90 | 4.20 |
| | Time | 20.96 | 23.20 | 24.65 | 26.61 | 16.48 | 15.88 | 22.18 | 27.30 | 17.01 | 24.73 | 6.93 | | | |
| 20 | Success / Fail | S | S | S | S | S | S | S | S | S | S | S | 100.00 | 15.88 | 3.95 |
| | Time | 15.40 | 10.94 | 14.07 | 12.93 | 11.74 | 15.35 | 23.66 | 13.00 | 19.53 | 19.14 | 18.96 | | | |
| 21 | Success / Fail | S | S | S | S | S | S | S | S | S | S | S | 100.00 | 25.15 | 9.77 |
| | Time | 25.62 | 21.00 | 48.43 | 29.74 | 36.48 | 17.54 | 19.58 | 17.30 | 16.36 | 23.26 | 21.29 | | | |
| 22 | Success / Fail | S | S | S | S | S | S | S | S | S | S | S | 100.00 | 15.86 | 3.20 |
| | Time | 13.39 | 17.31 | 15.47 | 13.20 | 11.04 | 14.20 | 17.50 | 18.29 | 21.43 | 13.06 | 19.57 | | | |
| 23 | Success / Fail | S | S | S | S | S | S | S | S | S | S | S | 100.00 | 18.24 | 4.99 |
| | Time | 20.90 | 15.42 | 11.14 | 22.74 | 16.77 | 11.32 | 20.50 | 13.59 | 22.15 | 19.54 | 26.59 | | | |

**Table D** Detailed results of the ring transfer task when using the virtual vision platform in the surgeon group

| No. | Indicator | Number of Ring | | | | | | | | | | | Success rate (%) | Time for transferring a ring | |
|---|---|---|---|---|---|---|---|---|---|---|---|---|---|---|---|
| | | 1 | 2 | 3 | 4 | 5 | 6 | 7 | 8 | 9 | 10 | 11 | | Mean | SD |
| 1 | Success / Fail | S | S | S | S | F | S | S | S | S | S | S | 90.91 | 26.84 | 12.74 |
| | Time | 54.22 | 40.12 | 20.62 | 13.99 | 14.65 | 23.19 | 15.91 | 36.77 | 19.76 | 21.86 | 21.94 | | | |
| 2 | Success / Fail | S | S | S | S | S | S | S | S | S | S | S | 100.00 | 26.94 | 10.08 |
| | Time | 25.82 | 51.37 | 38.14 | 31.27 | 23.09 | 19.50 | 15.56 | 24.84 | 21.30 | 23.99 | 21.45 | | | |
| 3 | Success / Fail | S | S | S | S | S | S | S | S | S | S | F | 90.91 | 38.95 | 10.71 |
| | Time | 51.44 | 38.70 | 60.67 | 25.72 | 44.51 | 38.08 | 34.88 | 29.30 | 29.89 | 36.32 | 36.27 | | | |
| 4 | Success / Fail | S | S | S | S | F | F | S | F | F | S | F | 54.55 | 33.19 | 12.89 |
| | Time | 17.06 | 19.58 | 40.55 | 47.35 | 12.10 | 19.84 | 30.34 | 117.38 | 33.83 | 44.26 | 22.23 | | | |
| 5 | Success / Fail | S | S | S | S | S | S | S | S | S | S | S | 100.00 | 26.69 | 8.17 |
| | Time | 29.76 | 35.56 | 20.86 | 41.18 | 22.96 | 31.93 | 33.99 | 18.13 | 21.84 | 21.65 | 15.78 | | | |
| 6 | Success / Fail | S | S | S | S | S | S | S | S | F | S | S | 90.91 | 35.53 | 11.58 |
| | Time | 30.21 | 51.14 | 53.54 | 25.58 | 20.72 | 38.95 | 31.00 | 45.09 | 24.67 | 36.50 | 22.59 | | | |
| 7 | Success / Fail | F | S | S | S | S | S | S | S | F | F | S | 72.73 | 44.12 | 15.80 |
| | Time | 59.37 | 42.23 | 38.03 | 43.35 | 27.16 | 27.30 | 75.78 | 43.43 | 39.35 | 58.80 | 55.64 | | | |
| 8 | Success / Fail | S | S | S | S | S | S | S | S | S | S | S | 100.00 | 39.54 | 4.70 |
| | Time | 31.84 | 42.63 | 36.02 | 37.32 | 40..82 | 37.74 | 36.00 | 44.42 | 38.50 | 46.39 | 44.57 | | | |
| 9 | Success / Fail | F | S | F | F | F | S | S | S | S | S | S | 63.64 | 35.13 | 10.33 |
| | Time | 18.41 | 35.12 | 9.35 | 26.38 | 81.88 | 34.51 | 35.63 | 53.87 | 37.34 | 30.50 | 18.94 | | | |
| 10 | Success / Fail | S | S | S | S | S | S | F | S | S | S | S | 90.91 | 20.59 | 8.80 |
| | Time | 16.54 | 17.96 | 16.86 | 17.32 | 12.52 | 28.36 | 8.56 | 12.19 | 22.79 | 19.60 | 41.75 | | | |
| 11 | Success / Fail | S | S | S | S | S | S | F | S | S | S | S | 90.91 | 22.49 | 6.03 |

| | | | | | | | | | | | | | | | |
|---|---|---|---|---|---|---|---|---|---|---|---|---|---|---|---|
| | Time | 23.41 | 20.38 | 24.67 | 28.23 | 17.18 | 14.32 | 15.88 | 14.88 | 31.07 | 20.81 | 29.90 | | | |
| 12 | Success / Fail | S | S | S | S | S | S | S | S | S | S | S | 100.00 | 28.74 | 10.54 |
| | Time | 34.04 | 21.75 | 17.19 | 22.55 | 38.62 | 33.08 | 18.68 | 30.78 | 17.14 | 31.18 | 51.10 | | | |
| 13 | Success / Fail | S | S | S | F | S | S | S | S | S | S | S | 90.91 | 20.41 | 2.88 |
| | Time | 19.27 | 20.46 | 15.87 | 6.96 | 26.33 | 19.08 | 19.65 | 20.55 | 18.90 | 24.02 | 19.98 | | | |
| 14 | Success / Fail | S | S | S | S | S | S | S | S | S | S | S | 100.00 | 35.12 | 7.86 |
| | Time | 48.86 | 35.24 | 27.26 | 42.14 | 35.37 | 31.86 | 20.09 | 43.02 | 35.87 | 30.96 | 35.69 | | | |
| 15 | Success / Fail | S | S | S | S | S | S | S | S | S | S | S | 100.00 | 27.95 | 12.88 |
| | Time | 28.19 | 31.21 | 18.44 | 20.67 | 18.39 | 58.79 | 18.19 | 25.69 | 20.39 | 22.85 | 44.61 | | | |
| 16 | Success / Fail | S | S | S | S | S | S | S | S | S | S | F | 90.91 | 23.88 | 5.28 |
| | Time | 13.57 | 26.01 | 20.87 | 30.88 | 26.67 | 26.34 | 16.65 | 26.30 | 24.41 | 27.06 | 39.82 | | | |
| 17 | Success / Fail | S | F | S | F | S | S | F | S | S | S | S | 72.73 | 29.89 | 15.11 |
| | Time | 20.18 | 35.79 | 65.36 | 26.30 | 25.42 | 29.54 | 11.13 | 24.86 | 30.96 | 26.68 | 16.15 | | | |
| 18 | Success / Fail | S | F | S | S | F | S | S | S | S | S | S | 81.82 | 25.80 | 10.58 |
| | Time | 31.30 | 31.39 | 17.23 | 22.34 | 13.09 | 50.49 | 19.33 | 18.90 | 30.84 | 18.98 | 22.78 | | | |
| 19 | Success / Fail | F | S | S | S | F | S | S | S | S | S | S | 81.82 | 31.59 | 14.98 |
| | Time | 10.88 | 20.73 | 20.18 | 29.30 | 39.74 | 32.10 | 26.05 | 57.83 | 55.62 | 25.19 | 17.27 | | | |
| 20 | Success / Fail | S | S | S | S | S | S | S | S | F | S | F | 81.82 | 20.65 | 9.58 |
| | Time | 15.91 | 37.06 | 11.27 | 16.87 | 18.40 | 14.11 | 19.28 | 15.90 | 7.43 | 37.01 | 12.38 | | | |
| 21 | Success / Fail | S | S | S | S | S | S | S | S | S | S | S | 100.00 | 23.54 | 6.19 |
| | Time | 24.59 | 28.48 | 18.83 | 17.34 | 36.69 | 21.06 | 30.00 | 25.19 | 18.05 | 21.42 | 17.26 | | | |
| 22 | Success / Fail | S | S | S | S | S | F | S | F | S | S | S | 81.82 | 33.00 | 10.16 |
| | Time | 22.47 | 34.62 | 28.43 | 30.14 | 39.78 | 8.79 | 22.58 | 25.64 | 55.30 | 28.09 | 35.58 | | | |
| 23 | Success / Fail | S | S | S | S | S | S | S | S | S | S | S | 100.00 | 22.08 | 4.02 |
| | Time | 15.98 | 29.94 | 25.43 | 26.96 | 21.34 | 21.28 | 22.31 | 21.73 | 18.82 | 18.37 | 20.72 | | | |

**Table E** Detailed results of the time for performing the scenarios when using the stereo viewer (DVC: DICOM viewer check, VSC: vital sign check)

| Participant No. | Novice | | | | Surgeon | | | |
|---|---|---|---|---|---|---|---|---|
| | DVC | VSC | Mean | SD | DVC | VSC | Mean | SD |
| 1 | 33.10 | 25.20 | 29.15 | 5.59 | 27.92 | 18.37 | 23.15 | 6.75 |
| 2 | 31.78 | 30.70 | 31.24 | 0.76 | 26.82 | 16.82 | 21.82 | 7.07 |
| 3 | 26.77 | 16.58 | 21.68 | 7.21 | 33.46 | 17.35 | 25.41 | 11.39 |
| 4 | 31.29 | 18.03 | 24.66 | 9.38 | 29.38 | 18.40 | 23.89 | 7.76 |
| 5 | 27.00 | 16.43 | 21.72 | 7.47 | 28.71 | 16.05 | 22.38 | 8.95 |
| 6 | 26.42 | 15.79 | 21.11 | 7.52 | 29.58 | 17.67 | 23.63 | 8.42 |
| 7 | 28.12 | 15.52 | 21.82 | 8.91 | 28.30 | 20.59 | 24.45 | 5.45 |
| 8 | 29.82 | 15.60 | 22.71 | 10.06 | 27.29 | 17.57 | 22.43 | 6.87 |
| 9 | 29.87 | 17.10 | 23.49 | 9.03 | 26.74 | 17.46 | 22.10 | 6.56 |
| 10 | 28.50 | 19.60 | 24.05 | 6.29 | 27.90 | 16.51 | 22.21 | 8.05 |
| 11 | 25.59 | 15.90 | 20.75 | 6.85 | 29.93 | 17.67 | 23.80 | 8.67 |
| 12 | 28.81 | 17.78 | 23.30 | 7.80 | 27.46 | 22.78 | 25.12 | 3.31 |
| 13 | 27.73 | 18.48 | 23.11 | 6.54 | 26.10 | 17.37 | 21.74 | 6.17 |
| 14 | 29.34 | 16.71 | 23.03 | 8.93 | 28.06 | 17.44 | 22.75 | 7.51 |
| 15 | 28.75 | 17.63 | 23.19 | 7.86 | 25.81 | 18.06 | 21.94 | 5.48 |
| 16 | 27.59 | 17.06 | 22.33 | 7.45 | 27.59 | 17.09 | 22.34 | 7.42 |
| 17 | 28.01 | 18.42 | 23.22 | 6.78 | 25.67 | 17.17 | 21.42 | 6.01 |
| 18 | 25.31 | 16.67 | 20.99 | 6.11 | 28.20 | 16.07 | 22.14 | 8.58 |
| 19 | 27.83 | 16.23 | 22.03 | 8.20 | 27.11 | 17.09 | 22.10 | 7.09 |
| 20 | 21.89 | 15.08 | 18.49 | 4.82 | 26.10 | 18.08 | 22.09 | 5.67 |
| 21 | 27.10 | 16.88 | 21.99 | 7.23 | 28.79 | 17.54 | 23.17 | 7.95 |

| | | | | | | | | |
|---|---|---|---|---|---|---|---|---|
| 22 | 28.00 | 17.62 | 22.81 | 7.34 | 26.05 | 17.40 | 21.73 | 6.12 |
| 23 | 29.43 | 18.26 | 23.85 | 7.90 | 27.03 | 16.69 | 21.86 | 7.31 |
| 24 | 29.97 | 16.14 | 23.06 | 9.78 | | | | |
| 25 | 27.30 | 15.98 | 21.64 | 8.00 | | | | |
| 26 | 31.04 | 17.66 | 24.35 | 9.46 | | | | |
| 27 | 26.26 | 17.01 | 21.64 | 6.54 | | | | |
| 28 | 26.92 | 18.66 | 22.79 | 5.84 | | | | |
| 29 | 26.94 | 17.50 | 22.22 | 6.68 | | | | |
| 30 | 27.95 | 17.39 | 22.67 | 7.47 | | | | |

**Table F** Detailed results of the time for performing the scenarios when using the virtual vision platform (DVC: DICOM viewer check, VSC: vital sign check)

| Participant No. | Novice | | | | Surgeon | | | |
|---|---|---|---|---|---|---|---|---|
| | DVC | VSC | Mean | SD | DVC | VSC | Mean | SD |
| 1 | 36.87 | 16.93 | 26.90 | 14.10 | 26.86 | 18.03 | 22.45 | 6.24 |
| 2 | 39.70 | 18.99 | 29.35 | 14.64 | 26.23 | 17.39 | 21.81 | 6.25 |
| 3 | 27.89 | 17.00 | 22.45 | 7.70 | 27.03 | 17.82 | 22.43 | 6.51 |
| 4 | 34.39 | 17.99 | 26.19 | 11.60 | 28.50 | 17.40 | 22.95 | 7.85 |
| 5 | 33.60 | 16.16 | 24.88 | 12.33 | 35.61 | 15.56 | 25.59 | 14.18 |
| 6 | 27.70 | 17.37 | 22.54 | 7.30 | 30.38 | 18.26 | 24.32 | 8.57 |
| 7 | 37.12 | 17.61 | 27.37 | 13.80 | 29.00 | 17.29 | 23.15 | 8.28 |
| 8 | 27.08 | 19.68 | 23.38 | 5.23 | 29.08 | 17.94 | 23.51 | 7.88 |
| 9 | 30.09 | 18.61 | 24.35 | 8.12 | 29.62 | 17.75 | 23.69 | 8.39 |
| 10 | 29.26 | 18.68 | 23.97 | 7.48 | 26.08 | 17.21 | 21.65 | 6.27 |
| 11 | 27.80 | 16.60 | 22.20 | 7.92 | 27.95 | 16.80 | 22.38 | 7.88 |
| 12 | 28.91 | 17.80 | 23.36 | 7.86 | 27.42 | 18.17 | 22.80 | 6.54 |
| 13 | 28.10 | 17.92 | 23.01 | 7.20 | 27.53 | 15.30 | 21.42 | 8.65 |
| 14 | 30.39 | 16.92 | 23.66 | 9.52 | 28.02 | 16.78 | 22.40 | 7.95 |
| 15 | 29.11 | 19.07 | 24.09 | 7.10 | 25.16 | 17.22 | 21.19 | 5.61 |
| 16 | 28.87 | 18.47 | 23.67 | 7.35 | 28.05 | 17.15 | 22.60 | 7.71 |
| 17 | 30.15 | 18.48 | 24.32 | 8.25 | 28.14 | 17.56 | 22.85 | 7.48 |
| 18 | 30.06 | 16.82 | 23.44 | 9.36 | 31.01 | 16.81 | 23.91 | 10.04 |
| 19 | 26.35 | 15.87 | 21.11 | 7.41 | 27.91 | 16.83 | 22.37 | 7.83 |
| 20 | 28.82 | 18.45 | 23.64 | 7.33 | 29.03 | 17.01 | 23.02 | 8.50 |
| 21 | 26.54 | 17.15 | 21.85 | 6.64 | 27.70 | 18.40 | 23.05 | 6.58 |

| | | | | | | | | |
|---|---|---|---|---|---|---|---|---|
| 22 | 30.26 | 16.65 | 23.46 | 9.62 | 27.70 | 17.93 | 22.82 | 6.91 |
| 23 | 28.04 | 17.07 | 22.56 | 7.76 | 26.01 | 18.11 | 22.06 | 5.59 |
| 24 | 28.58 | 16.66 | 22.62 | 8.43 | | | | |
| 25 | 29.08 | 15.65 | 22.37 | 9.50 | | | | |
| 26 | 29.07 | 19.97 | 24.52 | 6.43 | | | | |
| 27 | 25.19 | 16.96 | 21.08 | 5.82 | | | | |
| 28 | 27.85 | 17.51 | 22.68 | 7.31 | | | | |
| 29 | 27.74 | 17.01 | 22.38 | 7.59 | | | | |
| 30 | 27.55 | 18.37 | 22.96 | 6.49 | | | | |
